# Case report of fatal myocarditis with toripalimab and axitinib combination therapy

**DOI:** 10.1097/MD.0000000000046439

**Published:** 2025-12-26

**Authors:** Zhengxin Liu, Jinyu Tian, Shengjie Zeng, Chuan Liu

**Affiliations:** a Department of Urology, The Second Affiliated Hospital of Chongqing Medical University, Chongqing, China.

**Keywords:** axitinib, ICI-associated myocarditis, immune checkpoint inhibitor, immunotherapy, toripalimab

## Abstract

**Rationale:**

Cardiac immune-related adverse events (irAEs) are rare but potentially life-threatening complications of immune checkpoint inhibitor (ICI) therapy, with myocarditis associated with particularly high mortality even under aggressive treatment.

**Patient concerns:**

A 65-year-old male with advanced clear cell renal cell carcinoma and liver metastasis presented with progressive wheezing and fatigue following 4 cycles of combination therapy with toripalimab and axitinib, symptoms that failed to respond to conventional supportive measures.

**Diagnoses:**

Grade 4 ICI-associated myocarditis was confirmed through a combination of clinical manifestations, characteristic echocardiographic abnormalities, elevated cardiac biomarkers, and the exclusion of infectious causes via comprehensive serological evaluation.

**Interventions:**

The patient received multimodal immunosuppressive and supportive therapy, including high-dose methylprednisolone, intravenous immunoglobulin (IVIG), venoarterial extracorporeal membrane oxygenation (VA-ECMO), and therapeutic plasma exchange. Concurrently, broad-spectrum antibiotics were administered based on continuous microbial surveillance.

**Outcomes:**

Although transient hemodynamic stabilization was achieved, the patient subsequently developed severe nosocomial infections and progressive multiorgan failure, leading to death on day 24 of hospitalization.

**Lessons:**

This case underscores the necessity for early recognition and intensive monitoring in managing high-grade ICI-associated myocarditis. It further highlights the critical role of infection prevention strategies, particularly the implementation of microbial surveillance-guided antibiotic prophylaxis, in patients undergoing combination immunotherapy regimens.

**Plain Language Summary:**

We report a 65-year-old male with advanced renal cell carcinoma who developed ICI-associated myocarditis following 4 cycles of combination immunotherapy. The patient presented with progressive dyspnea and fatigue, necessitating hospitalization. Comprehensive diagnostic evaluation confirmed grade 4 myocarditis based on clinical features, elevated cardiac biomarkers, echocardiographic abnormalities, and exclusion of infectious causes. Treatment consisted of multimodal immunosuppressive and life-support therapies, including high-dose methylprednisolone, intravenous IVIG, therapeutic plasma exchange, VA-ECMO, and empiric broad-spectrum antibiotics guided by microbial surveillance. Despite transient hemodynamic stabilization, the patient ultimately progressed to multiorgan failure and died on day 24 of hospitalization. This case underscores the life-threatening nature of ICI-associated myocarditis and highlights the critical need for early recognition, aggressive supportive management, and proactive anti-infective prophylaxis in patients receiving combination immunotherapy.

## 1. Introduction

Toripalimab in combination with axitinib has been shown to significantly improve both progression-free survival and overall survival as a first-line treatment for patients with advanced clear cell renal cell carcinoma (ccRCC) of intermediate or poor risk. However, cardiac immune-related adverse events (irAEs) associated with this regimen remain underreported. Notably, immune checkpoint inhibitor (ICI)-associated myocarditis is frequently associated with a poor prognosis, with mortality rates reported between 39.7% and 50%.^[1]^ Early recognition and intervention are therefore critical.

## 2. Case presentation

### 2.1. Initial presentation

A 65-year-old male was diagnosed with advanced ccRCC with liver metastasis and initiated first-line combination therapy consisting of toripalimab (240 mg every 3 weeks) and axitinib (5 mg twice daily). His medical history included hypertension, which was well controlled with levamlodipine besylate and irbesartan/hydrochlorothiazide. After completion of 4 treatment cycles, the patient was hospitalized due to progressive wheezing and fatigue. Vital signs upon admission were stable, and there were no clinical signs or laboratory evidence of active infection.

### 2.2. Diagnostic workup

Electrocardiogram revealed sinus tachycardia, low voltage in the limb leads, and mild ST-segment elevation. Cardiac biomarkers were elevated, with increased creatine kinase-MB levels. Echocardiography showed a reduced left ventricular ejection fraction of 31%, new-onset pericardial effusion, and dilated right ventricular dimensions compared to prior imaging. Comprehensive serological testing for common viral and bacterial pathogens – including major respiratory viruses, herpesviruses, blood-borne viruses, and selected spirochetal and rickettsial agents – yielded negative results. Coronary computed tomography angiography excluded acute coronary syndrome. Based on the National Cancer Institute Common Terminology Criteria for Adverse Events version 5.0 and the 2023 Cardio-Oncology Guidelines from the Chinese Society of Clinical Oncology, a diagnosis of grade 4 ICI-associated myocarditis was established. Consequently, both toripalimab and axitinib were permanently discontinued.

### 2.3. Management

The patient was transferred to the intensive care unit (ICU) for multidisciplinary management. High-dose methylprednisolone (1 g/day) and intravenous intravenous immunoglobulin (IVIG) (20 g/day) were initiated promptly. Given hemodynamic instability, venoarterial extracorporeal membrane oxygenation (VA-ECMO) was instituted on hospital day 2. Table 1 summarizes the patient’s laboratory findings at the time of hospital admission and ICU transfer.

**Table 1:** Laboratory findings on admission and being admitted to the ICU.

Event	At admission	At ICU	Reference range
Leukocytes (10^9^)	7.12	7.29	3.50–9.50
Erythrocytes (10^12^)	4.65	4.12	4.35–5.80
Hemoglobin (g/L)	137	126	130–175
Platelets (10^9^)	112	25	100–300
PRO-BNP (ng/L)	16,351.0	15,905.0	NA
Glomerular filtration rate (mL/min)	73.9	23.7	80.0–300.0
Creatine phosphokinase (U/L)	48.1	22.0	38.0–174.0
Creatine kinase-MB (U/L)	18.8	25.5	0.0–24.0
Lactate dehydrogenase (U/L)	/	1551	120–246
Myohemoglobin (ng/mL)	42.8	59.33	0.00–70.00
cTnI (ng/mL)	0.297	0.192	0.000–0.030
Alanine aminotransferase (U/L)	594	1319	9–50
Aspartate aminotransferase (U/L)	419	1833	15–40
Albumin (g/L)	32.1	32.5	40.0–55.0
Immunoglobulin G (g/L)	/	17.60	7.00–16.00
Immunoglobulin A (g/L)	/	1.43	70–4.00
Immunoglobulin M (g/L)	/	0.48	0.40–2.30
Ig-κ (g/L)	/	3.98	1.70–3.70
Ig-λ (g/L)	/	2.11	0.90–1.80
Complement C3 (g/L)	/	0.44	0.90–1.80
Complement C4 (g/L)	/	0.14	0.10–0.40
Alkaline phosphatase (U/L)	80	78	45–125
Cholinesterase (KU/L)	4.95	3.80	5.00–12.00
Urea nitrogen (mmol/L)	9.58	16.68	3.60–9.50
Serum creatinine (μmol/L)	93.5	244	71.0–133.0
D-Dime (ng/mL)	/	1840.9	0.0–550.0
C-reactive protein (mg/L)	/	20.78	0.00–10.00
Procalcitonin (ng/mL)	/	1.2488	0.0000–0.0500
IL-2 (pg/mL)	/	0.71	≤7.50
IL-4 (pg/mL)	/	<1.57	≤16.50
IL-6 (pg/mL)	/	182.28	≤5.40
IL-10 (pg/mL)	/	63.98	≤12.90
TNF-α (pg/mL)	/	<1.57	≤16.50
IFN-γ (pg/mL)	/	<1.66	≤23.10
CD45^+^ (cells/μL)	/	780	1230–3700
CD3^+^ %	/	72.54	50.62–85.22
CD3^+^ # (cells/μL)	/	566	520–2860
CD3^+^CD4^+^ %	/	18.21	20.17–58.99
CD3^+^CD4^+^ # (cells/μL)	/	142	400–1610
CD3^+^CD8^+^ %	/	48.48	8.99–36.72
CD3^+^CD8^+^ # (cells/μL)	/	378	150–1250
CD4/CD8	/	0.4	1.4–2.0

# = count, CD = cluster of differentiation, ICU = intensive care unit, IFN-γ = interferon-γ, Ig-κ = immunoglobulin-kappa light chain, Ig-λ = immunoglobulin-lambda light chain, IL = interleukin, NA = not available, PRO-BNP = pro-B type natriuretic peptide, TNF-α = tumor necrosis factor-α.

On ICU day 4, methylprednisolone was tapered to 500 mg/day, and IVIG was discontinued owing to declining troponin I levels. Sputum culture identified Enterobacter cloacae; cefoperazone-sodium sulbactam was administered based on antimicrobial susceptibility testing. Protective isolation measures were implemented to minimize infection risk.

### 2.4. Clinical course

**Figure 1.**
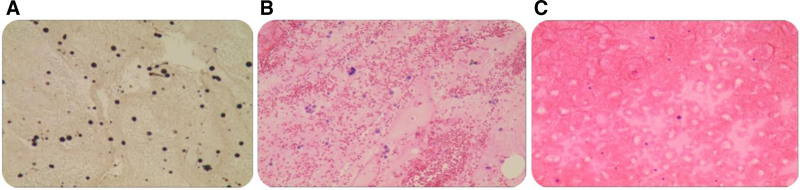
Pericardial fluid cytology: sparse mesothelial cells and lymphocytes. Immunocytochemical staining: CK (+), CK7 (+), EMA (+), MelanA (−), Vim (+), PAX-2 (−), CD10 (−), Ki-67 (+), LCA (+), CK5/6 (+), CR (++), CK20 (−), CD68 (−).

**Figure 2.**
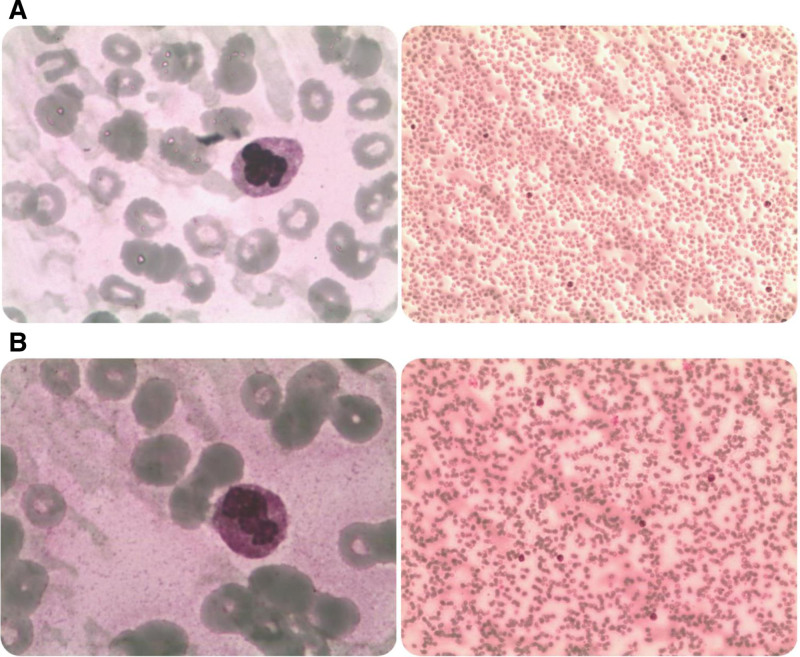
(A) Bone marrow aspirate smear: mature granulocytes show a small number of toxic granules. Mature erythrocytes exhibit mild anisocytosis, and polychromatophilic erythrocytes are present. Scattered platelets are infrequently observed. Lymphocytes constitute 1% with normal morphology. No parasites identified. (B) Bone marrow aspirate smear: neutrophilic granulocytosis with left shift, marked toxic granulation (++++), and cytoplasmic vacuolation (++). Mature red blood cells show anisocytosis, with 5% metarubricytes. Platelets are sparsely distributed. Lymphocytopenia is evident. No parasites detected.

After 10 days of VA-ECMO, cardiac function showed improvement, allowing for device weaning and removal. Pericardial fluid analysis (Fig. 1) revealed sparse mesothelial cells and lymphocytic infiltration. Hematologic evaluation demonstrated concurrent depletion of erythrocytes, lymphocytes, and platelets. Bone marrow aspiration (Fig. 2A) showed toxic granules in mature granulocytes and markedly reduced platelets.

On ICU day 13, plasma exchange (2000 mL/day) was initiated due to persistent lack of myocardial recovery. The patient subsequently developed hypotension and intermittent fever, requiring vasopressor support with norepinephrine and inotropic support with dobutamine. Antibiotic therapy was escalated to meropenem. Blood cultures grew Enterococcus faecium, prompting addition of vancomycin. Methylprednisolone was maintained at 120 mg daily to balance immunosuppression with infection risk.

Given suspicious urinary findings suggestive of fungal infection, caspofungin was initiated. By hospital day 20, the patient exhibited worsening renal function and hepatic impairment classified as Child-Pugh class B. Consequently, vancomycin dosage was reduced, and methylprednisolone was tapered to one-third of the prior dose. Concurrently, oxygen saturation declined progressively, necessitating endotracheal intubation and mechanical ventilation. Despite administration of red blood cell and platelet transfusions, thrombopoietic agents, and granulocyte colony-stimulating factor, bone marrow suppression persisted without improvement. Repeat bone marrow aspiration revealed granulocytosis with left-shifted nuclear maturation, anisocytosis among mature erythrocytes, and lymphocytopenia (Fig. 2B). The patient’s condition deteriorated into multiorgan failure, ultimately resulting in death on hospital day 24.

**Figure 3.**
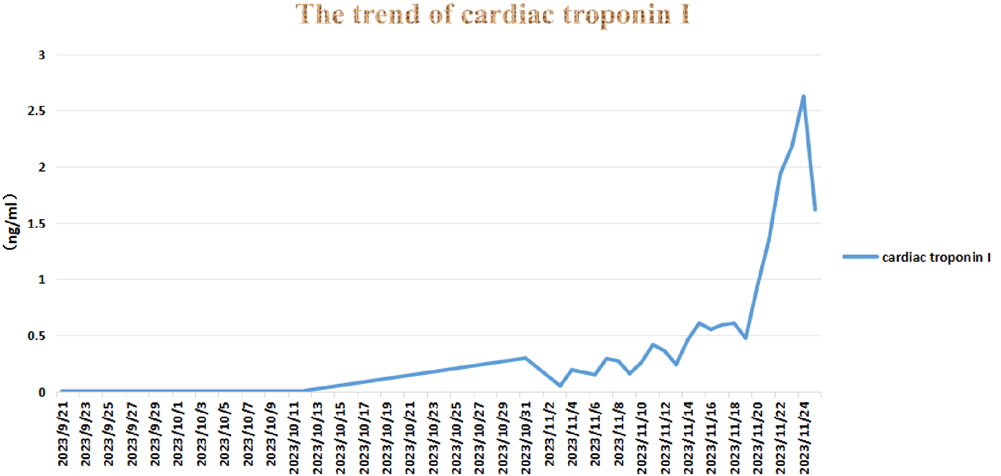
Serial measurements of cardiac troponin I over time.

Serial measurements of cardiac troponin I throughout the clinical course are summarized in Figure 3.

## 3. Discussion

Anti-programmed cell death-ligand 1 (PD-L1) monoclonal antibodies have transformed cancer therapy across multiple tumor types. As a newly developed PD-L1 inhibitor, toripalimab is widely used in China for the treatment of advanced malignancies.^[2]^ According to the phase III RENOTORCH study, toripalimab plus axitinib demonstrated significant and clinically meaningful improvements in progression-free survival and overall response rate compared with sunitinib as first-line therapy in patients with advanced ccRCC of intermediate or poor risk.^[3]^ However, the adverse effects of ICI cannot be overlooked, among which cardiac irAEs are the most life-threatening, including myocarditis, pericardial disease, and supraventricular or ventricular arrhythmias. Although the incidence of ICI-associated myocarditis is low, ranging from 0.41 to 1.33%, it is associated with high fatality rates.^[1]^

Nine cases of adverse events associated with toripalimab were reported in previous studies (Table 2).^[4–11]^ All patients were Chinese citizens. Table 2 summarizes their clinical characteristics, diagnoses, and treatments. Patient ages ranged from 33 to 69 years, with 5 females (55.6%). Notably, only 3 cases were associated with cardiac irAEs; one patient had a history of hypertension, and treatments such as methylprednisolone were effective. All patients had advanced cancer or distant metastases and received toripalimab. Furthermore, endomyocardial biopsy remains the gold standard for diagnosing ICI-associated myocarditis. However, in most patients – including those reported in Table 2 – myocarditis can be diagnosed based on compatible symptoms, laboratory findings, electrocardiogram, and cardiac magnetic resonance imaging, with biopsy reserved for selected cases. ICI-associated myocarditis developed within 4 to 12 weeks in both the patients presented in the table and our own cohort, consistent with previously reported onset times in the literature.^[12]^ However, apart from our patient, all other patients with ICI-associated myocarditis listed in the table received early-stage antibiotics to prevent infection.

**Table 2:** Clinical characteristics of cases.

Study	Gender/Age	History of cardiovascular disease	Symptoms	Medication guidelines	Time*	Adverse events	Treatments	Diagnostic method	Death
Chen Y^[4]^ (2022)	Female/43	NA	Dyspnea, chest pain with palpitation, generalized rash and malaise	Mixed liposarcoma of the right upper extremity with bilateral lower extremity metastases	8	Myocarditis, myositis, Hashimoto’s thyroiditis, skin toxicity	IVMP (200 mg/d), oral levothyroxine replacement therapy	Symptom, laboratory tests, ECG, coronary angiography, left heart catheterization, echocardiography, thyroid ultrasound, chest CT	No
Chen Y^[4]^ (2022)	Female/64	Hypertension	Chest tightness and malaise	Pancreatic adenocarcinoma with multiple metastases (liver, adrenal glands, and multiple lymph nodes)	10	Myocarditis, ICIs-associated pneumonia	IVMP (200 mg/d), IVIG (20 g/d for 3 d)	Symptom, laboratory tests, ECG, echocardiography, chest CT, lung biopsy	No
Luo YB^[5]^ (2021)	Female/47	NA	Progressive diplopia, myalgia, limb weakness, dysphagia, dyspnea	Type B2 thymoma with bone metastasis	4	Myositis, myocarditis, myasthenia gravis	IVMP (500 mg/d for 1 d, 250 mg/d for 5 d), PSL (60 mg/d for 4 wk), temporal PM, mechanical ventilator support, noninvasive ventilation, intramuscular neostigmine, IVIG (0.4 g/kg/d for 5 d)	Symptom, laboratory tests, ECG, electromyography	No
Zhou H^[6]^ (2022)	Female/63	NA	Severe vertigo, vomiting, nystagmus, cerebellar ataxia, cognitive impairment	Metastatic melanoma	The day after first toripalimab injection	Anti-GAD65-associated autoimmune encephalitis	IVIG (0.4 mg/kg/d for 5 d), PLEX	Symptom, laboratory tests, electroencephalography, head MRI	No
Lin Y^[7]^ (2022)	Male/69	NA	NA	Metastatic squamous cell lung carcinoma	18	Sarcoid/granulomatous-like reaction	Follow-up observations	Symptom, laboratory tests, abdominal contrast enhanced CT, hepatic ultrasound, hepatic contrast enhanced MRI, liver nodule	No
Li X^[8]^ (2021)	Female/38	NA	Rashes, shortness of breath, edema of both lower limbs, mild swelling of oropharyngeal mucosa with repeated bleeding and scabbing	Metastatic cervical cancer	4	Severe bullous skin reactions	IVMP (80 mg/d), IVIG (0.4 g/kg/d), diphenhydramine, cardiotonic agents, Diuretic agents, vasodilator agents, blood transfusion, antibiotics	Symptom, laboratory tests	Yes
Fan Y^[9]^ (2023)	Male/58	NA	Urinary frequency, urinary urgency, urodynia, gross hematuria	Advanced metastatic liver cancer	1	Cystitis associated with ICIs	Antibiotics, IVMP (120 mg/d for 5 d, 80 mg/d for 3 d), PSL (40 mg/d)	Symptom, laboratory tests, contrast-enhanced CT, pathologies of bladder mucosa, urine culture tests	Yes
Zhou Y^[10]^ (2023)	Male/65	NA	Fatigue, a sudden syncopal attack	Hypopharyngeal squamous cell carcinoma with the cervical lymph nodes recurred	1	Cytokine-release syndrome	Venoclysis, supplemental oxygen, filgrastim, dexamethasone	Symptom, laboratory tests, cranial diffusion-weighted imaging	Yes
Wang Y^[11]^ (2024)	Male/33	NA	NA	Ewing sarcoma	24	Endocrine gland insufficiency	Discontinue ICI therapy, initiate hormone replacement therapy	Laboratory tests, pituitary MRI	No

CT = computed tomography, d = day, ICU = intensive care unit, IVIG = intravenous immunoglobulin, IVMP = intravenous methylprednisolone, MRI = magnetic resonance imaging, NA = not available, PLEX = plasma exchange, PM = pacemaker, PSL = prednisolone, w = week.

* Time between initiation of PD-1 inhibitors and irAEs (wk).

In the present case, pericardiocentesis findings were associated with tumor pericardial metastasis, raising the possibility that the cardiac manifestations could have been attributable to malignancy; however, this could not be definitively ruled out. The patient’s relatives declined autopsy, and therefore, more definitive pathological information could not be obtained. Nevertheless, most cases of cardiac metastases are clinically silent and are typically diagnosed postmortem. The most common cancer types associated with cardiac metastases include lung cancer, breast cancer, and hematologic malignancies.^[13]^

According to the National Cancer Institute Common Terminology Criteria for Adverse Events, immunotherapy should be permanently discontinued in the event of any grade 3 or 4 cardiac irAEs.^[14]^ The American Society of Clinical Oncology clinical practice guidelines also recommend withholding immunotherapy in patients with myocarditis and initiating high-dose corticosteroids, such as 1 to 2 mg/kg per day of oral prednisone or intravenous methylprednisolone, depending on symptom severity, for all grades of toxicity.^[15]^ The patient’s ICI-associated myocarditis was promptly detected and treated with high-dose methylprednisolone, intravenous IVIG, anti-infective therapy, VA-ECMO, and plasma exchange. Although cardiac function improved on the 10th day of VA-ECMO support, this improvement was not sustained. Based on the clinical course and the patient’s incomplete response to immunosuppressive therapy, it is reasonable to conclude that the patient had hormone-resistant ICI-associated myocarditis. Furthermore, the recurrence of cardiac dysfunction was likely attributable to infection-induced exacerbation of myocardial injury. Given the findings from 2 sequential bone marrow aspirations, prolonged high-dose methylprednisolone therapy may have contributed to profound immunosuppression. Combined with invasive interventions such as VA-ECMO, the patient faced an elevated risk of infection. Despite timely escalation of antimicrobial therapy, septic shock ensued; these infections may have further deepened the immunosuppressed state, leading to rapid clinical deterioration. Current European Society of Cardiology guidelines emphasize the need to exclude infectious etiologies before initiating immunosuppressive therapy in suspected cases of myocarditis, particularly in patients presenting with systemic symptoms or underlying immunosuppression.^[16]^ Nevertheless, a scarcity of systematic data on the microbial profile associated with myocarditis limits the evidence base for choosing targeted prophylactic antibiotics. In light of this, early empirical broad-spectrum antibiotic therapy – initiated when autoimmune myocarditis is suspected and before steroid use – followed by tailored adjustments according to subsequent microbiological culture results and infection biomarkers, could contribute to improved prognostic outcomes.

Notably, in current clinical practice, many healthcare providers remain unaware of the importance of monitoring infection-related markers and initiating prophylactic antibiotics during the early management of ICI-associated myocarditis – a gap that may compromise treatment success. Broad-spectrum antibiotics may be considered for early empiric anti-infective coverage, with subsequent adjustments based on body fluid cultures, antimicrobial susceptibility testing, infection markers, and temperature trends. Antibiotic regimens should be promptly tailored according to susceptibility results, with careful attention to preventing the emergence of multidrug-resistant organisms.

The patient had a history of antihypertensive medication use with well-controlled blood pressure. The potential interaction between antihypertensive drugs and cardiac irAEs during combined PD-L1 and VEGFR inhibitor therapy warrants further investigation. Serial troponin I monitoring may enhance early detection, as monthly assessments proved inadequate in this case.^[6,17]^

## 4. Article highlights

- Toripalimab plus axitinib offers clinical benefit in advanced ccRCC but carries risk of fatal cardiac irAEs.
- Elevated troponin levels during immunotherapy should raise suspicion for autoimmune myocarditis. Early initiation of prophylactic anti-infective therapy is recommended.
- A 65-year-old male with ccRCC and liver metastases developed grade 4 ICI-associated myocarditis after 4 cycles of toripalimab and axitinib. Despite aggressive multimodal treatment, he died of infection-related multiorgan failure.
- This case highlights the severity of cardiac irAEs and underscores the need for further research into improved diagnostic and therapeutic strategies.

## Acknowledgments

The authors gratefully acknowledge the authorization and support provided by the patients and their families. This study received no specific grant from any funding agency in the public, commercial, or not-for-profit sectors. The authors declare that they have no competing interests.

## Author contributions

**Conceptualization:** Zhengxin Liu.

**Data curation:** Zhengxin Liu.

**Formal analysis:** Zhengxin Liu.

**Investigation:** Zhengxin Liu.

**Methodology:** Zhengxin Liu.

**Project administration:** Chuan Liu.

**Resources:** Jinyu Tian.

**Supervision:** Chuan Liu.

**Validation:** Shenjie Zeng.

**Visualization:** Zhengxin Liu.

**Writing – original draft:** Zhengxin Liu.

**Writing – review & editing:** Chuan Liu.
